# Altered manganese and iron biomarkers in welders: a multi-element biomonitoring study across blood, serum and urine

**DOI:** 10.3389/fpubh.2026.1850447

**Published:** 2026-06-15

**Authors:** Johannes Fischer, Anne Nagel, Nick Erdmann, Andy Schmied

**Affiliations:** Federal Institute for Occupational Safety and Health (BAuA), Berlin, Germany

**Keywords:** ferritin, gas metal arc welding, human biomonitoring, iron, manganese, metal homeostasis, occupational exposure, welding fume

## Abstract

**Background:**

Interpreting biomonitoring data for welding fume exposure remains challenging due to complex metal mixtures and tight homeostatic regulation of essential elements. In gas metal arc welding (GMAW), manganese is of particular concern due to its neurotoxic potential upon overload. Iron, although being the dominant mass fraction of welding fumes, has received less attention despite possible disturbance of systemic metal homeostasis.

**Methods:**

Twelve GMAW welders and matched controls were investigated in this pilot study using a multi-matrix biomonitoring approach. Blood and serum samples were collected pre- and post-shift, and urine samples post-shift only. 17 metals were quantified by inductively coupled plasma tandem mass spectrometry (ICP-MS/MS). Iron-related biomarkers (ferritin, transferrin saturation, soluble transferrin receptor (sTfR)) were additionally assessed.

**Results:**

Urinary chromium, iron, manganese, antimony, and vanadium were elevated in welders compared with controls. Blood manganese, although still widely used in occupational biomonitoring, showed only minor differences between groups, consistent with well-known concerns about its limited sensitivity as a marker of internal exposure. In contrast, serum and urinary manganese demonstrated more distinct patterns between welders and controls. Descriptive subgroup analysis according to respiratory protection use suggested higher biomarker concentrations in welders without powered air-purifying respirators. Welders exhibited approximately threefold higher serum ferritin levels accompanied by reduced sTfR, unaltered serum iron, and increased urinary iron excretion. This biomarker pattern is compatible with alterations in systemic iron homeostasis affecting iron storage rather than circulating iron pools. Ferritin concentrations were positively associated with manganese in serum and urine.

**Conclusion:**

These findings highlight the complexity of biomonitoring in welding environments and suggest limitations of single-matrix approaches for assessing internal manganese and iron exposure under contemporary working conditions. Multi-matrix strategies and further studies integrating biological and exposure-related measurements are needed to improve occupational biomonitoring concepts for welders.

## 1 Introduction

Welding is one of the most common industrial fabrication techniques worldwide, employing about 11 million professional welders and around 110 million additional workers with welding-related exposures (1, 2). From an occupational health perspective, welding is considered a high-risk activity. Workers are exposed to ultraviolet radiation, noise, ergonomic strain, and welding fume. Welding fume is generated when metal is heated to high temperatures during the welding process, leading to vaporization of metal constituents that subsequently condense into ultrafine respirable particles. Depending on the materials and process parameters, the fume may contain aluminum, cadmium, chromium, copper, iron, lead, manganese, molybdenum, nickel, zinc, and other elements (3). The International Agency for Research on Cancer classified welding fume as carcinogenic for humans (Group 1) (1).

Gas Metal Arc Welding (GMAW) is among the most frequently used welding processes, widely applied in construction, manufacturing, and shipbuilding. In this process, a wire electrode is continuously fed through a welding torch, where it melts under an electric arc onto the workpiece. The weld pool is protected from atmospheric contamination by a shielding gas, typically argon, helium, carbon dioxide, or their mixtures. Compared to other welding methods, GMAW produces relatively high amounts of welding fume (4–6). The exact composition strongly varies with the process parameters, material, and working conditions (7, 8).

Iron is the dominant component of welding fumes produced during GMAW on mild steel, accounting for around 80% of the total fume mass, mainly as oxides such as Fe_2_O_3_ and Fe_3_O_4_ formed when vaporized metal rapidly oxidizes in air (3, 7, 9). The resulting particles are largely in the submicron range and can penetrate deep into the alveolar region of the lung. Although iron is considered less toxic than other metals, chronic inhalation of iron-rich fume can cause local accumulation of insoluble particles in the lung, a condition known as pulmonary siderosis (10). Furthermore, inflammation and oxidative stress have been observed after exposure to iron-rich welding fume (11–13). Despite its quantitative dominance in welding fume, the systemic bioavailability and biomonitoring relevance of inhaled iron remain insufficiently understood (14, 15). This uncertainty complicates the interpretation of iron-related biomarkers in exposed populations.

Among the metals present in GMAW fumes, manganese is of particular toxicological relevance and concern. Although an essential trace element, it becomes neurotoxic at elevated exposure levels (16). It is added to steel and welding consumables as a deoxidizer and alloying element and readily vaporizes in the welding arc (17). The resulting manganese oxides form fine particles contributing to the respirable fraction of the welding fume (17, 18). After inhalation, a proportion of manganese is available for pulmonary uptake and can enter systemic circulation and reach the central nervous system (14, 17, 19). Its transport and cellular uptake are tightly regulated and largely rely on shared pathways like Ca^2+^ channels, Zn transporters of the SLC39 family, and most notably transferrin dependent and independent process via divalent metal transporter 1 (DMT1) (20, 21). Because manganese utilizes these common transport systems, it competes with other essential metals such as Fe, Zn, and Co for cellular uptake and systemic distribution. Importantly, these pathways are not restricted to essential elements only. Non-essential divalent metals such as Cd, Pb, and Ni are also transported via DMT1 (22). Consequently, welding fume exposure may not only increase internal manganese levels but also alter the systemic distribution and regulation of other metals through competition at shared transport and regulatory sites. Such interactions may contribute to broader disturbances of metal homeostasis rather than isolated changes in individual metal concentrations (23, 24).

Chronic manganese overexposure is associated with a neurological disorder with clinical features resembling Parkinson’s disease, often referred to as “manganism.” It is characterized by bradykinesia, gait dysfunction, and dystonia (25, 26). At the cellular level, Mn disrupts dopaminergic neurotransmission including dopamine release, uptake, and storage (27).

To mitigate the risk to manganese-exposed workers, many countries have introduced occupational exposure limits and guideline values. For example, the German occupational exposure limit as well as the threshold limit value recommended by the American Conference of Governmental Industrial Hygienists are both set at 0.02 mg/m^3^ for respirable manganese (28, 29). However, compliance with these limits is often not achieved under real-world working conditions as multiple studies indicate (4, 5, 30). Consequently, personal protective measures like powered air-purifying respirators (PAPR) have been more widely implemented in recent years. These devices maintain a positive pressure of filtered air within the helmets of welders, minimizing particle inhalation substantially when applied correctly (31). However, their effectiveness in reducing systemic metal uptake under routine working conditions requires evaluation. In this context, reliable assessment of internal exposure becomes increasingly important.

Biomonitoring is an important tool to assess internal metal exposure of welders and to evaluate the effectiveness of such protective measures. Unlike air sampling, it aims to reflect the amount of substance actually absorbed into the body by measuring metal concentrations in a suitable biological matrix. The choice of matrix, commonly whole blood, serum, or urine, depends on the metal’s distribution kinetics, transport mechanisms, and primary pathways of elimination. In addition to exposure assessment, multi-element biomonitoring across matrices may help to identify alterations in metal homeostasis and inter-metal relationships resulting from complex mixture exposures. Timing of sample collection in relation to the exposure window, as well as interactions between different metals, such as competitive absorption or shared transport pathways need to be taken into account (32–34). For metals that are subject to tight homeostatic control, biomarker choice and interpretation are particularly complex. To date, manganese is usually assessed in whole blood although several studies indicate that measured concentrations do not reliably reflect airborne exposure (34, 35). In the case of iron, the mere measurement of total concentrations in serum or urine alone may not show the whole picture. Instead, other biomarkers like ferritin, transferrin saturation and soluble transferrin receptor (sTfR) can be altered in welders and might be of additional interest for biomonitoring (32, 33, 36).

This pilot study provides a comprehensive and up-to-date assessment of internal metal exposure in a cohort of GMAW welders by quantifying up to 17 metals across whole blood, serum, and urine. In addition, protein-based biomarkers involved in metal transport and regulation were systematically evaluated. Particular emphasis was placed on manganese and iron as key constituents of GMAW fume. Both metals are subject to tight homeostatic regulation, making exposure assessment and biomarker interpretation considerably more complex than for other metals. Multi-matrix biomonitoring studies of this scope remain limited, especially those integrating classical metal measurements with regulatory proteins. By assessing pre- and post-shift differences in welders and comparing them with a non-exposed control group, this study seeks to broaden knowledge of the complex interrelationships of potential biomarkers and possibly identify starting points for further research to improve and refine biomonitoring strategies in occupational health.

## 2 Materials and methods

### 2.1 Study design and participants

The present pilot study was conducted in 2023 and 2024 among professional GMAW welders exposed to welding fume and a non-exposed control group matched by age and sex. Recruitment was open to individuals of all sexes; however only, male participants fulfilled the inclusion criteria and consented to participate during the study period. Due to the application of predefined exclusion criteria and exclusion of welders with insufficient welding activity on the sampling day, the final study population differed slightly between groups and consisted of 12 welders (mean age 40 years, range 26–59) and 14 controls (mean age 41 years, range 25–64). Whole blood and serum samples were collected before and after the work shift and a post-shift urine sample was obtained on the same day. In welders, sampling took place on a Monday during a regular workweek; controls were sampled on a comparable weekday. Welders were recruited from four different companies across Germany predominantly processing mild steel. Included subjects were active full-time GMAW welders with an average welding experience of 19 years (range 1–40 years). Six of them used powered air-purifying respirators (PAPR) on the day of sample collection. Demographic data, general health status, and work history were collected through a structured interview. In addition, participants completed a questionnaire documenting their daily work tasks and occupational activities.

Exclusion criteria included anemia, impaired liver or kidney function, acute inflammation (C-reactive protein (CRP) above 5.5 mg/L), severe obesity (body mass index greater than 35), and bleeding disorders or anticoagulant therapy. The study was conducted in accordance with relevant national law and institutional guidelines. The study was approved by the Ethics Committee of the Berlin Chamber of Physicians in April 2022 (approval number Eth-14/22). All participants provided written informed consent prior to enrolment. Samples and data were pseudonymized prior to analysis.

### 2.2 Collection and handling of biological samples

Blood samples were collected before and after the shift. The venipuncture site was cleaned and sterilized prior to blood collection. Blood was drawn from an antecubital vein, first collecting two 8.5 mL samples into gel separator tubes for analysis of serum proteins. Afterwards, two 6 mL samples were collected into trace element-certified BD Vacutainer tubes (BD, Franklin Lakes, NJ, United States) to be used for metal analysis in serum. Lastly, one 6 mL sample was collected in a K_2_-EDTA BD Vacutainer for the determination of trace elements in whole blood. After clotting, serum was separated by centrifugation at 1300 × g for 10 min.

Spot urine samples were collected after the work shift in 500 mL polyethylene (PE) containers. All collection containers were pre-cleaned to avoid trace metal contamination. All whole blood, serum, and urine samples intended for metal analysis were immediately aliquoted into 15 mL metal-free centrifuge tubes (Labcon, Petaluma, CA, United States) and frozen. Samples were transported at −20 °C to the laboratories of the Federal Institute for Occupational Safety and Health (BAuA) in Berlin, where they were stored at −80 °C and subsequently analyzed. Serum samples intended for protein determination were transported and stored at 2–8 °C and analyzed within 2 days of collection.

### 2.3 Analytical methods

Metal and metalloid concentrations in urine, serum and blood samples were determined by inductively coupled plasma tandem mass spectrometry (ICP-MS/MS) using an Agilent 8,900 ICP-MS/MS (Agilent Technologies, Waldbronn, Germany).

Al, Ba, Be, Bi, Cd, Co, Cr, Cu, Fe, Li, Mn, Mo, Ni, Pb, Sb, Sr, and V were analyzed in urine samples after acidic dilution as described previously with minor modifications (37).

For the determination of Co, Cu, Fe, Mn and Zn in serum and Cd, Co, Cu, Hg, Mn, Pb and Zn in blood, specimens were diluted 10-fold with an alkaline solution containing 1 g/L EDTA, 0.5 g/L Triton X-100, 0.25% (V/V) ammonia and 1% (V/V) propan-2-ol. A matrix-matched calibration was carried out for all biological specimen types. Instrumental conditions and the applied limits of quantification are reported in Supplementary Tables S1, S2. Commercially available materials from RECIPE (Munich, Germany) and from Sero AS (Billingstad, Norway) were used for quality control.

Creatinine concentration in urine was measured photometrically according to the Jaffé method (38). Ferritin, transferrin (Tf), and soluble transferrin receptor (sTfR) concentrations were determined by an accredited external diagnostics laboratory via electrochemiluminescence immunoassay, turbidimetric immunoassay and turbidimetry, respectively. Transferrin saturation (TfS) was calculated from the ratio of serum iron (μg/L) and serum transferrin (mg/L), using a standard conversion factor of 70.9 and is expressed in percent.

### 2.4 Statistics

All statistical analyses were performed using SPSS Statistics 31.0 (IBM Corp., Armonk, NY, United States). To describe data distribution, the number of samples (N), number of samples below limit of quantification (N < LOQ), geometric mean (GM), median and (interquartile) range are reported for each analyte and group. Urinary concentrations are presented both as volume-based concentrations and creatinine-adjusted values. If more than 50% of values were above the LOQ, concentrations below the LOQ were imputed as LOQ/2 for statistical testing; otherwise, only descriptive statistics were reported. Correlations between post-shift biomarker concentrations were assessed using Spearman’s rank correlation coefficient (*ρ*). Given the exploratory nature and the limited sample size of this pilot study, no formal adjustment for multiple comparisons across all endpoints was applied. Therefore, statistical results should be interpreted as hypothesis-generating and are intended to support pattern identification rather than confirmatory inference. Group comparisons were conducted using the Mann–Whitney U test, and paired comparisons across sampling times were performed using the Wilcoxon signed-rank test. Statistical significance was defined as *α* = 0.05.

In accordance with the World Health Organization’s recommendation, only urine samples with creatinine concentrations within the reference range of 0.3–3.0 g/L were considered valid and included in the analysis (39).

## 3 Results

### 3.1 Urinary metal concentrations in welders and the control group

Urinary concentrations of Al, Ba, Be, Bi, Cd, Co, Cr, Cu, Fe, Li, Mn, Mo, Ni, Pb, Sb, Sr, and V were determined in professional welders and in a non-exposed control group at the end of a work shift. Comprehensive analytical results for all urinary metals, including detection frequencies, descriptive parameters (median, GM, IQR) and results of group comparisons, are provided in Supplementary Table S4. A selection of key biomarkers across matrices is summarized in Table 1. Median creatinine concentration in welders was 1.5 g/L (IQR 1.3–1.8 g/L) and in the control group 0.8 g/L (IQR 0.6–1.3 g/L). The urine sample of one welder was excluded from analysis due to a creatinine concentration outside the required range. Unless otherwise specified, urinary concentrations are presented as creatinine-adjusted values. Median urinary concentrations of Al, Bi, Cr, Fe, Mn, Mo, Sb, and V were higher in welders compared with controls. For Al, Bi, Cr, and Mn, concentrations in most control group samples were below the LOQ, whereas measurable concentrations were more frequently detected in welders, precluding formal statistical testing. This suggests a group-related difference in both detection frequency and concentration distributions. Large relative differences between groups were observed for Fe and Sb, with median concentrations in welders exceeding those of controls by approximately 4.4-fold and 6.4-fold, respectively. No significant differences were observed for Ba, Cd, Co, Cu, Li, Ni, Pb, and Sr. For Be, all samples from both welders and controls were below the LOQ. Correlation analysis among welders revealed strong positive associations between urinary Mn and V (Spearman’s *ρ* = 0.897, *p* < 0.001), as well as urinary Mn and Cr (ρ = 0.807, *p* = 0.003). Scatterplots are presented in the Supplementary Figures S1A,B. No corresponding correlations were observed in the control group.

**Table 1:** Median concentrations of selected metals and proteins in urine (U), blood (B) and serum (S) taken post-shift (pre-shift Mn(B) and Mn(S) are included to enable comparison, full dataset available in Table S3).

Parameter	Unit	Welders		Controls		*p*
		N/N < LOQ	Median	N/N < LOQ	Median	
Co(U)	μg/g cr.	11/0	0.12	14/0	0.23	0.066
Cr(U)	μg/g cr.	11/0	0.23	14/8	0.074*	n.a.
Fe(U)	μg/g cr.	11/0	13.9	14/0	3.16	**<0.001**
Mn(U)	μg/g cr.	11/3	0.09	14/14	0.05*	n.a.
Mo(U)	μg/g cr.	11/0	27.3	14/0	42.0	**0.018**
Ni(U)	μg/g cr.	11/0	0.78	14/0	1.24	0.051
Sb(U)	μg/g cr.	11/0	0.18	14/3	0.028	**<0.001**
V(U)	μg/g cr.	11/0	0.056	14/4	0.027	**<0.001**
Co(B)	μg/L	12/0	0.102	13/0	0.136	**0.016**
Mn(B) pre-shift	μg/L	12/0	10.0	14/0	7.85	0.06
Mn(B)	μg/L	12/0	10.1	13/0	8.22	0.098
Pb(B)	μg/L	12/0	15.9	13/0	11.2	0.41
Zn(B)	μg/L	12/0	6,170	13/0	6,820	**0.035**
Co(S)	μg/L	12/0	0.133	13/0	0.188	**0.011**
Fe(S)	μg/L	12/0	860	13/0	916	0.81
Ferritin(S)	μg/L	12/0	303	13/0	98	**0.008**
Mn(S) pre-shift	μg/L	12/0	0.51	14/0	0.45	**0.009**
Mn(S)	μg/L	12/0	0.55	13/0	0.45	**<0.001**
sTfR(S)	mg/L	12/0	2.27	14/0	2.52	**0.046**

Urinary concentrations are presented as creatinine-adjusted values [μg/g cr.]. Stars indicate parameters for which median volume-based urinary concentration was below LOQ. Significant *p*-values are given in bold.

### 3.2 Blood metal concentrations in welders and the control group

**Figure 1:**
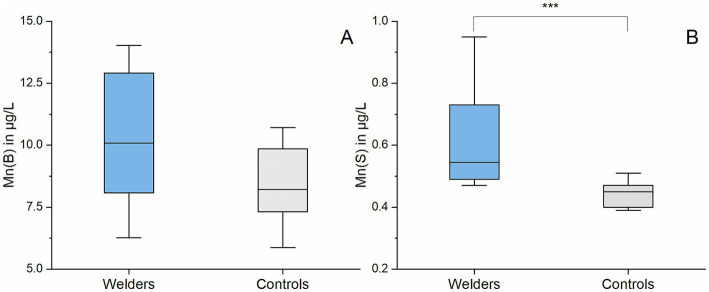
Concentrations of manganese in blood samples **(A)** and in serum samples **(B)** of welders and controls. The boxes and boundaries of the whiskers represent the 25th/75th and 10th/90th percentiles, respectively. The horizontal lines indicate the median values. ***: significant difference, *p* < 0.001.

Blood concentrations of Cd, Co, Cu, Hg, Mn, Pb and Zn were determined in welders and controls before and after the work shift. In one case, the post-shift blood/serum sample from a control participant was not obtained. Complete analytical results for all blood metals at both sampling times are presented in Supplementary Tables S3, S4. Selected blood metal concentrations are summarized in Table 1. Across both sampling times, all blood samples were above the LOQ for all elements except Hg(B), where one sample in each group was below the LOQ. Overall, measured blood metal concentrations showed little variation between pre-shift and post-shift samples in both groups. At pre-shift, no statistically significant differences in concentrations were observed between welders and controls (Figure 1A). Mn(B) tended to be slightly higher in welders, whereas Co(B) and Zn(B) tended to be lower. At post-shift, Co(B) and Zn(B) concentrations again were slightly lower and now reaching statistical significance.

### 3.3 Serum metal concentrations in welders and the control group

**Figure 2:**
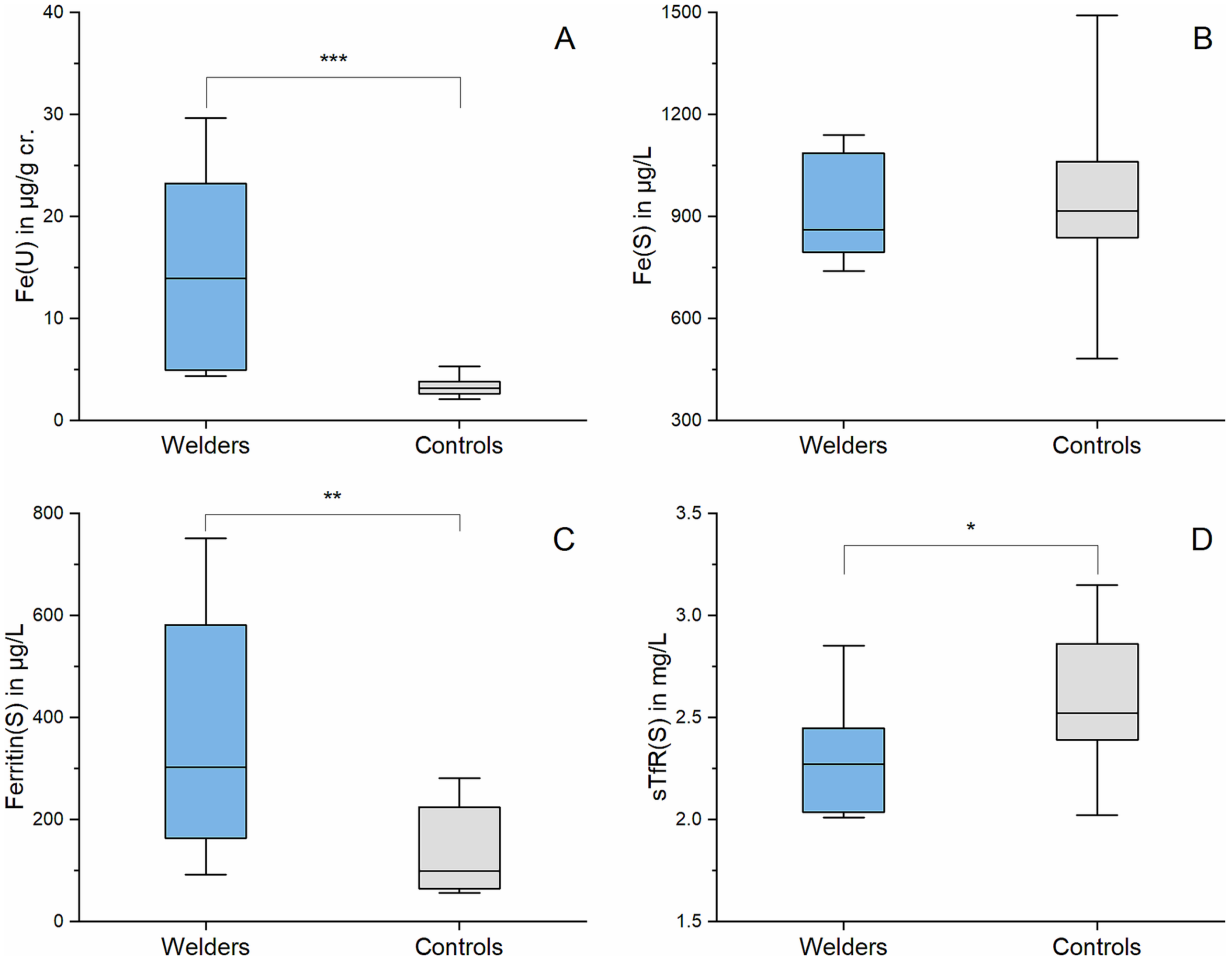
Concentrations of iron in urine samples **(A)** and iron **(B)**, ferritin **(C)**, and sTfR **(D)** in serum samples of welders and controls. The boxes and boundaries of the whiskers represent the 25th/75th and 10th/90th percentiles, respectively. The horizontal lines indicate the median values. ***: significant difference, *p* < 0.001. **: significant difference, *p* < 0.01. *: significant difference, *p* < 0.05.

Serum concentrations of Co, Cu, Fe, Mn, and Zn were determined in welders and controls before and after the work shift. Complete analytical results for all serum metals at both sampling times are presented in Supplementary Tables S3, S4. All serum samples were above the LOQ for all investigated elements. Overall, serum metal concentrations showed little variation between pre-shift and post-shift samples in both groups. An exception was observed for Mn(S), which increased slightly but significantly across the work shift among welders (median 0.51 μg/L to 0.55 μg/L, *p* = 0.004), whereas median concentrations in controls remained unchanged at 0.45 μg/L over the day (Figure 1B). No significant differences in Fe(S) were observed between welders and controls at either sampling time (Figure 2B). At post-shift, Co(S) was significantly lower in welders compared with controls (median 0.13 μg/L vs. 0.19 μg/L, *p* = 0.011). No statistically significant group differences were observed for Cu(S) or Zn(S) at either sampling time.

### 3.4 Further manganese- and iron-related parameters

Ferritin, soluble transferrin receptor (sTfR), and transferrin (Tf) concentrations as well as transferrin saturation (TfS) were determined in serum samples of welders and controls before and after the work shift. Detailed results for all iron-related biomarkers at both sampling times are presented in Supplementary Tables S3, S4. Selected iron status parameters included in Table 1 are shown to facilitate cross-matrix comparison. Overall, serum iron status parameters showed little variation between pre-shift and post-shift samples within both groups. In contrast, pronounced group differences were observed for ferritin concentrations. Median ferritin levels were substantially higher in welders, exceeding control medians by about threefold at both sampling times. Post-shift median ferritin concentrations were 303 μg/L in welders and 98 μg/L in controls (*p* = 0.008, Figure 2C). In addition, post-shift sTfR concentrations were slightly but significantly lower in welders compared with controls (median 2.27 mg/L vs. 2.52 mg/L, *p* = 0.046; Figure 2D). No statistically significant group differences were observed for Tf or TfS at either sampling time. Median CRP levels were 1.55 mg/L (IQR 0.83–2.3 mg/L) in welders and 0.8 mg/L (0.3–1.3 mg/L) in controls. Correlation analysis among welders revealed several significant associations within and across matrices. Serum ferritin concentrations were associated with Mn(S) (*ρ* = 0.671, *p* = 0.017), Mn(U) (ρ = 0.771, *p* = 0.006), and V(U) (ρ = 0.866, *p* = 0.001). Furthermore, Mn(S) was positively correlated with Mn(U) (ρ = 0.798, *p* = 0.003). Corresponding scatterplots are presented in Supplementary Figures S1C–F.

### 3.5 Subgroup analysis by PAPR usage

**Figure 3:**
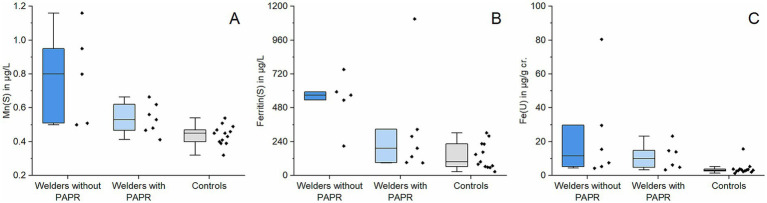
Concentrations of manganese **(A)** and ferritin **(B)** in serum samples and iron in urine samples **(C)** of welders with and without PAPR compared to the concentrations found in samples of the control group. The boxes represent the interquartile range (IQR). Boundaries of the whiskers are based on the 1.5 IQR value.

Since the use of powered air-purifying respirators (PAPR) during welding tasks may influence biomonitoring results, welders were grouped accordingly and biomarker concentrations were compared descriptively. Comprehensive descriptive results for all previously mentioned parameters stratified by respiratory protection status are provided in the Supplementary Table S5. Selected biomarkers illustrating the observed concentration patterns are summarized in Table 2. Given the small subgroup sizes, the subgroup analysis was considered exploratory and interpreted only descriptively. Therefore, no formal statistical comparisons between respiratory protection subgroups were performed. Across the parameters presented in Table 2, a consistent gradient was observed, with the highest concentrations in welders without respiratory protection, intermediate concentrations in welders using PAPR, and the lowest concentrations in the control group. This pattern is illustrated for selected biomarkers in Figure 3.

**Table 2:** Measured concentrations of selected metals and ferritin in urine (U), blood (B) and serum (S) taken post-shift depending on the use of powered air-purifying respirators (PAPR).

Parameter	Unit	Welders without PAPR		Welders with PAPR		Controls	
		N/N < LOQ	Median	N/N < LOQ	Median	N/N < LOQ	Median
Bi(U)	μg/g cr.	5/0	0.162	6/1	0.022	14/10	0.005*
Cr(U)	μg/g cr.	5/0	0.29	6/0	0.23	14/8	0.074*
Fe(U)	μg/g cr.	5/0	15.5	6/0	10.1	14/1	3.16
Mn(U)	μg/g cr.	5/0	0.41	6/3	0.08	14/14	0.05*
Sb(U)	μg/g cr.	5/0	0.22	6/0	0.17	14/3	0.028
V(U)	μg/g cr.	5/0	0.087	6/0	0.045	14/4	0.027
Mn(B)	μg/L	5/0	11.82	7/0	8.19	13/0	8.22
Mn(S)	μg/L	5/0	0.8	7/0	0.53	13/0	0.45
Ferritin(S)	μg/L	5/0	570	7/0	193	13/0	98

Urinary concentrations are presented as creatinine-adjusted. Stars indicate median volume-based urinary concentrations below LOQ.

## 4 Discussion

### 4.1 Comparison of biomonitoring data in welders and controls

Accurate interpretation of human metal biomonitoring data requires careful attention to methodological details, as even minor external contamination or procedural inconsistencies can substantially bias results, particularly for trace elements. To address this, all blood and serum samples were collected using trace metal-certified materials, and the first serum tube from each participant was not used for elemental analysis to avoid artificially elevated metal concentrations caused by initial contact with collection equipment. Blood, serum, and urinary metal concentrations in the non-exposed control group were generally within background exposure range and consistent with published reference data for northern Germany as well as current German Biological Reference Values (BAR) (detailed comparison in Supplementary Table S6) (40, 41). This confirms both the validity of the control group and the analytical approach. Accordingly, no statistically significant differences between welders and controls were observed for urinary Ba, Cd, Co, Cu, Li, Ni, Pb, Sr, and Be. While some of these elements may occur in welding fumes depending on materials and process conditions, they are generally not regarded as dominant constituents of GMAW fumes, which is consistent with the absence of clear group differences in the present study. In contrast, welders showed higher post-shift urinary concentrations of Cr, Fe, Mn, Sb, and V, consistent with the known composition of welding fume and previous biomonitoring studies. Ellingsen et al. (42) also reported significantly higher median urinary concentrations of Cr (1.1 vs. 0.11 μ/g cr.) and V (0.08 vs. 0.05 μg/g cr.) in shipyard welders compared with a non-exposed control group (42). Similarly, Rafiee et al. reported higher geometric mean urinary concentrations of Cr, Fe, Mn and V in professional Canadian welders than in controls (43). Both studies indicate similar trends to the results presented here. Although elevated urinary Sb concentrations have not been reported in these studies and Sb has generally been investigated less frequently in biomonitoring studies of welders, the increase observed in the present study appears plausible since Sb has been identified as a constituent of welding fumes before (8, 44). In addition, the observed correlations between urinary Mn and V as well as urinary Mn and Cr are in line with previous reports suggesting co-variation of these metals may occur in welding fume exposure (42, 45).

In contrast, whole blood and serum metal concentrations showed only minor differences between welders and controls and little variation between pre- and post-shift sampling times. Depending on the specific element and its toxicokinetic properties, blood and serum concentrations may show limited responsiveness to short-term exposure changes, whereas urinary metal concentrations often reflect more recent uptake (46). Biomarkers for welding fume exposure are influenced by multiple potential confounders, including differences in welding techniques, base materials, consumables, and workplace conditions (47). In addition, element- and matrix-specific differences are expected due to distinct toxicokinetic properties and biological half-lives (46). To improve comparability across participants, all welder samples were collected on a Monday following at least 2 days without welding activities. While this approach reduces short-term exposure variability, it does not fully eliminate differences in cumulative exposure patterns. No personal air monitoring data were available; therefore, external exposure could not be quantified and biomarker interpretation is limited to internal exposure patterns.

### 4.2 Manganese biomonitoring

Manganese biomonitoring is of particular relevance in occupational welding settings due to its neurotoxic effect. Chronic inhalation of manganese-containing welding fumes has been associated with oxidative stress and neurological impairments resembling Parkinsonian syndromes referred to as “manganism” (25, 48, 49). Accordingly, a wide range of biological matrices has been investigated for Mn biomonitoring, including whole blood, red blood cells, serum or plasma, saliva, urine, hair, fingernails and toenails, each reflecting different exposure windows and toxicokinetic characteristics and yielding heterogeneous results across studies (24, 34, 45, 47, 50–55). Among these matrices, Mn in whole blood has most frequently been applied in welding biomonitoring studies and occupational health examinations, primarily due to historical precedence and analytical feasibility. However, the suitability of Mn(B) as an exposure biomarker has been questioned due to its tight homeostatic control. Evidence from state-of-the-art welding workplaces has demonstrated a poor correlation between blood manganese concentrations and external exposure levels, which led to the withdrawal of the biological limit value for manganese in whole blood in Germany (35, 56). Nevertheless, blood Mn continues to be widely measured in welding studies and occupational health examinations, largely because no clearly superior alternative biomarker has yet been established.

In the present study, Mn concentrations in welders were elevated in blood, serum, and urine compared to the control group. Median post-shift blood manganese concentration in welders was slightly lower than those reported by Pesch et al. (5) in a German cohort of 241 GMAW welders (10.1 vs. 11.5 μg/L), while being somewhat higher than values reported by Ellingsen et al. (47) in a cohort of 96 male welders (geometric mean 10.1 vs. 8.6 μg/L) (5, 47). However, differences in Mn(B) concentrations between welders and controls in the present study were not statistically significant, and substantial overlap of concentration distributions between the groups was observed (Figure 1A). Furthermore, no relevant differences in Mn(B) concentrations between pre-shift and post-shift sampling were observed in this study, which is plausible given the slow manganese kinetics within the erythrocyte compartment and the limited exposure window of 1 day assessed in the present study. All welders were below the current German Biological Reference Values (BAR) for blood manganese of 15 μg/L (Supplementary Table S6) (40). Taken together, these findings indicate that, under the exposure conditions and within the study population investigated here, blood manganese may have limited suitability as a biomarker for both short- and long-term manganese exposure in welders.

Several biomonitoring studies have reported significantly higher serum manganese concentrations in welders compared with control groups, which is consistent with the findings of this study (Figure 1B) (24, 32, 55). For example, Ellingsen et al. (32) found geometric mean serum manganese concentrations in a cohort of 137 welders of 1.04 μg/L vs. 0.77 μg/L in non-welding referents (this study: 0.61 vs. 0.44 μg/L; see Supplementary Table S4) (32). In our study, a small but significant increase in Mn(S) from pre-shift to post-shift was observed among welders, whereas no corresponding change occurred in the control group. Compared to Mn(B), Mn(S) may therefore appear somewhat more responsive to short-term exposure. However, the observed difference was small and based solely on group-level differences rather than quantitative air exposure data. Furthermore, a consistent linear correlation between exposure intensity and Mn(S) has not yet been sufficiently demonstrated in the literature and remains questionable (53).

In the present study, post-shift urinary manganese concentrations were detectable in most welders, whereas all control samples were below the LOQ, precluding formal statistical comparison. Nevertheless, the observed distribution pattern suggests higher urinary manganese concentrations in welders and is consistent with findings from several previous studies (32, 43, 57). These results may support the interpretation of urinary manganese as an indicator of increased uptake following welding activities. However, as with Mn(S), a clear and direct correlation between external manganese exposure and urinary manganese concentrations has not been consistently demonstrated in the literature and remains questionable (53). The interpretation of urinary manganese is further complicated by toxicokinetic considerations. Manganese is predominantly eliminated via the biliary-fecal pathway, while urinary excretion represents only a minor route of elimination. Consequently, urinary manganese concentrations are typically low, particularly in non-exposed populations, and should therefore be interpreted with caution.

Overall, Mn(B) appeared to show limited sensitivity to welding-related exposure under the conditions of this study, whereas serum and urinary manganese demonstrated more distinct differences between welders and controls and may be more responsive to recent exposure. However, these findings given the limitations of each matrix, including homeostatic regulation, low excretion rates and analytical constraints, no single biomarker appears to be fully suitable, and a combined multi-matrix approach as well as further research remains necessary.

### 4.3 Iron status of welders

Iron, although representing the major mass fraction in GMAW welding fumes, has received considerably less attention in occupational biomonitoring than manganese and other metals with well-established neurotoxic or carcinogenic properties. Furthermore, lower solubility and bioavailability of inhaled iron compared to other welding fume constituents have been proposed (14, 15). However, chronic inhalation of iron-containing welding fumes has not only been associated with pulmonary siderosis in welders, but also oxidative stress and associations with lung and kidney cancer are discussed (11, 13, 58, 59).

The iron status findings of the present study are largely consistent with previous reports. Median serum ferritin concentrations were approximately threefold higher in welders compared with controls (Figure 2C). Elevated ferritin levels in welders have also been described by Ellingsen et al. (32) and Lu et al. (33), although with less pronounced differences, probably due to different exposure conditions (32, 33). Soluble transferrin receptor concentrations were modestly but significantly reduced in welders (Figure 2D), similarly as in the cohort of Lu et al. (33), although we could not observe the enhanced Fe(S) levels in welders vs. controls (Figure 2B) (33).

Overall, the combination of markedly elevated ferritin, slightly but significantly reduced sTfR, unchanged Fe(S) and TfS is compatible with an expansion of body iron stores rather than acute alteration in circulating iron. Ferritin primarily reflects stored iron, whereas Fe(S) and TfS represent circulating iron pools, readily available for cellular uptake, which remain unaltered (60, 61). In contrast, reduced sTfR in welders suggests decreased cellular iron demand and thereby increased intracellular iron availability, consistent with expanded storage (61). Together, this pattern supports the interpretation that welding fume exposure is associated with subclinical alterations in systemic iron homeostasis that predominantly affect iron storage rather than circulating iron compartments. Distortion of biomarkers (especially ferritin) due to overt systemic inflammatory conditions appears less likely, as all participants had CRP levels below 5.5 mg/L. However, low-grade inflammatory processes below this threshold cannot be completely ruled out, and oxidative stress-related ferritin induction may occur independently of systemic iron overload (62).

In addition to altered storage markers, urinary iron concentrations were approximately fourfold higher in welders compared to controls in our cohort (Figure 2A). Similar elevations have been reported in other occupational studies on GMAW and manual metal arc welders, though again with smaller effect sizes (32, 33). The presence of markedly increased urinary iron despite comparable serum iron levels may suggest a systemic redistribution of iron in order to eliminate excess absorbed iron via enhanced renal excretion, further supporting the presence of disturbed iron homeostasis. However, no direct evidence is generated in this study and further mechanistic research is required.

Interpretation of the observed iron-related alterations should consider that the present study included exclusively male participants. Iron status markers such as ferritin, TfS, and sTfR are known to differ substantially by sex due to physiological differences in iron handling and storage (63, 64). Consequently, the extent to which the present findings can be generalized to female welders remains uncertain. Future studies including larger and more diverse study populations are therefore needed to clarify potential sex-specific differences in iron homeostasis related to welding fume exposure.

The observed positive association between ferritin and serum manganese concentrations may reflect not only co-exposure but possibly also shared homeostatic regulation, although interpretation remains limited due to the small sample size and observational nature of the data. Iron and manganese utilize common transport and regulatory pathways, including DMT1 and transferrin-mediated uptake, supporting the hypothesis that shared transport and regulatory pathways may contribute to interactions between iron and manganese homeostasis in welders (24).

Interestingly, iron-related alterations in welders may also extend to metals that are not considered constituents of GMAW fumes. In the present study, welders exhibited consistently lower cobalt concentrations across blood, serum and urine compared with controls. Similar differences were observed by Ellingsen et al. (32) in a cohort of welders compared to referents and negative associations between Co(B), Co(S) and Co(U) and serum ferritin concentrations were described (32). While the underlying biological mechanisms remain unclear, strong inverse associations between iron status markers and cobalt excretion have already been described in 1975 by Sorbie et al. (65). These findings may suggest that changes in iron homeostasis induced by welding fume exposure may be associated with altered systemic kinetics and elimination of other divalent metals, even when they are not directly related to the exposure source. However, dietary differences between the groups may have influenced these results and a broader study approach would be needed to further investigate this topic.

Taken together, these results are consistent with a pattern in which chronic welding fume exposure is associated with subclinical alterations of iron homeostasis possibly characterized by increased storage, altered receptor expression, and enhanced urinary excretion of iron. Given the shared transport systems for several divalent metals, such alterations may extend beyond iron itself and influence the systemic handling of other metals. Iron, although often considered of secondary concern compared to manganese, may therefore warrant greater attention in occupational biomonitoring and risk assessment of welding fumes.

### 4.4 Effect of respiratory protection on biomonitoring results

Investigating the effectiveness of protection measures was not the primary objective of this study, and the subgroup analysis is strongly limited by the small number of welders in each category. Nevertheless, descriptive evaluation of the data showed a consistent concentration gradient, with generally higher values in welders without PAPR, intermediate values in welders using PAPR, and lower concentrations in the control group (Table 2, Figure 3). This pattern was particularly evident for several metals in urine and for manganese in blood, serum and urine. However, due to the exploratory nature of the subgroup analysis, no conclusions regarding the effectiveness of respiratory protection can be drawn from the present data. The observed descriptive trends are generally consistent with previous studies reporting lower welding fume concentrations in the breathing zones of welders using suitable PAPR, accompanied by lower urinary concentrations of multiple metals, including Cr, Mn, and V, lower Mn(B) concentrations, and reduced serum ferritin levels compared to welders without PAPR (36, 43, 66, 67). However, welders equipped with PAPR still exhibited higher metal concentrations than controls, indicating that respiratory protection reduces but not fully prevents systemic metal uptake. Possible contributing factors discussed in the literature and based on our observations in the field include reduced protective performance due to suboptimal fit of the equipment and inhalation of unfiltered ambient air during non-welding tasks or work breaks when the respiratory protection is temporarily removed or opened (66, 67). In addition, welding fumes may persist in the workplace environment for extended periods, particularly in facilities with insufficient ventilation, potentially contributing to background exposure beyond active welding periods. The absence of personal air monitoring data and the heterogeneous working conditions further limit interpretation of subgroup-related differences.

### 4.5 Limitations and further research

The present pilot study was designed to provide a comprehensive multi-matrix assessment of internal metal exposure under current real-world welding conditions. In line with this objective, the focus was placed on biological monitoring rather than on quantitative air exposure assessment. Personal air measurements were therefore not integrated into the present analysis, which limits the ability to directly relate internal concentrations to measured airborne exposure levels. Consequently, the present study does not allow quantitative exposure-response relationships between airborne metal concentrations and internal biomarker levels. Furthermore, only post-shift urinary samples were collected. Therefore, only group comparisons between welders and controls were possible, whereas individual within-day changes could not be assessed. This pilot study included a modest number of participants, which limits statistical power. This particularly affects subgroup analyses and correlation analyses, which should therefore be interpreted as exploratory and hypothesis-generating. The cohort reflects real-world diversity in welding tasks, base materials, consumables, and workplace settings, which naturally contributes to inter-individual differences in exposure and biomarker concentrations and should be considered when interpreting the results. While the study design does not capture long-term temporal dynamics or establish causality, it describes exposure-associated group differences between welders and controls under practical working conditions. Since all participants were male, the generalizability of our findings is limited, particularly with regard to sex-specific differences in iron-related biomarkers. Larger studies including more diverse participant populations are therefore needed.

The matrix-specific differences observed for manganese and iron-related markers underline the need for a more detailed characterization of their toxicokinetic and regulatory dynamics under real-world welding conditions. In particular, the combination of altered iron storage markers and correlations between ferritin and manganese may suggest potential shared regulatory pathways that require further investigation in larger studies. Moreover, total metal concentrations alone provide limited insight into biologically relevant species.

From a regulatory and occupational health perspective, the present findings further highlight the need to refine biomonitoring strategies for manganese. The limited discriminatory power of individual matrices and the influence of homeostatic regulation complicate the interpretation of measured concentrations in occupational medical practice. Under current conditions, biomarker results may therefore be difficult to translate into clear exposure or risk assessments at the individual level. More robust, exposure-responsive, and clinically interpretable biomarkers are needed to support reliable decision-making in routine occupational health examinations. The identification and validation of such markers remain an important objective for further research and require larger study collectives and concurrent exposure measurements.

## 5 Conclusion

This study provides an exploratory multi-matrix assessment of internal metal exposure in GMAW welders under current working conditions. Elevated urinary concentrations of several welding-related metals, particularly Cr, Fe, Mn, Sb, and V, indicate increased internal exposure compared with a matched non-exposed control group. In contrast, blood-based markers showed comparatively limited discriminatory power, likely reflecting homeostatic regulation of these metals and the influence of physiological variability.

In our pilot study, no single biological matrix appeared fully suitable for assessment of manganese exposure from welding fumes. While serum and urinary manganese may appear more responsive to recent exposure than whole blood, substantial overlap between groups and the known impact of physiological regulation limit their interpretability at the individual level, particularly in small-scale occupational studies. These findings highlight the need for integrated multi-matrix approaches and more sensitive biomarkers for occupational manganese monitoring.

With respect to iron, the combination of markedly elevated ferritin, reduced sTfR, and increased urinary iron is compatible with subclinical alterations in systemic iron homeostasis in welders. Given the shared transport and regulatory pathways of iron and manganese, welding fume exposure may be consistent with broader regulatory adaptations affecting multiple divalent metals.

Overall, welding fume exposure is associated with detectable changes in manganese and iron biomarkers across matrices. Current single-matrix approaches seem limited in their ability to support reliable individual risk interpretation under real-world working conditions, underscoring the need for more robust and physiologically informed biomonitoring strategies in occupational health.

## Data Availability

The original contributions presented in the study are included in the article/Supplementary material, further inquiries can be directed to the corresponding author/s.

## References

[1] IARC Working Group on the Evaluation of Carcinogenic Risks to Humans, International Agency for Research on Cancer. Welding, Molybdenum Trioxide, and Indium Tin Oxide. Lyon, France: International Agency for Research on Cancer World Health Organization (2018). p. 310. 31268644

[2] Guha N Loomis D Guyton KZ Grosse Y El Ghissassi F Bouvard V . Carcinogenicity of welding, molybdenum trioxide, and indium tin oxide. Lancet Oncol. (2017) 18:581–2. doi: 10.1016/S1470-2045(17)30255-3, 28408286

[3] Antonini JM. Health effects of welding. Crit Rev Toxicol. (2003) 33:61–103. doi: 10.1080/713611032 12585507

[4] Flynn MR Susi P. Manganese, iron, and total particulate exposures to welders. J Occup Environ Hyg. (2010) 7:115–26. doi: 10.1080/15459620903454600, 20013450

[5] Pesch B Weiss T Kendzia B Henry J Lehnert M Lotz A . Levels and predictors of airborne and internal exposure to manganese and iron among welders. J Expo Sci Environ Epidemiol. (2012) 22:291–8. doi: 10.1038/jes.2012.9, 22377681

[6] Lehnert M Pesch B Lotz A Pelzer J Kendzia B Gawrych K . Exposure to inhalable, respirable, and ultrafine particles in welding fume. Ann Occup Hyg. (2012) 56:557–67. doi: 10.1093/annhyg/mes025, 22539559 PMC3387834

[7] Zimmer AT Biswas P. Characterization of the aerosols resulting from arc welding processes. J Aerosol Sci. (2001) 32:993–1008. doi: 10.1016/S0021-8502(01)00035-0

[8] Dueck ME Rafiee A Mino J Nair SG Kamravaei S Pei L . Welding fume exposure and health risk assessment in a cohort of apprentice welders. Ann Work Expo Health. (2021) 65:775–88. doi: 10.1093/annweh/wxab016, 33889935

[9] Antonini JM Taylor MD Zimmer AT Roberts JR. Pulmonary responses to welding fumes: role of metal constituents. J Toxicol Environ Health A. (2004) 67:233–49. doi: 10.1080/15287390490266909, 14681078

[10] Patel RR Ryu JH Yi ES. Systemic Iron overload associated with welder’s Siderosis. Am J Med Sci. (2009) 337:57–9. doi: 10.1097/01.MAJ.0000308933.80112.49, 18941405

[11] Thanachoksawang C Navasumrit P Hunsonti P Chompoobut C Chaisatra K Autrup H . Exposure to airborne iron oxide nanoparticles induces oxidative DNA damage and inflammatory responses: a pilot study in welders and in human lung epithelial cell line. Toxicol Environ Heal Sci. (2022) 14:339–49. doi: 10.1007/s13530-022-00148-3

[12] Pesch B Lotz A Koch HM Marczynski B Casjens S Käfferlein HU . Oxidatively damaged guanosine in white blood cells and in urine of welders: associations with exposure to welding fumes and body iron stores. Arch Toxicol. (2015) 89:1257–69. doi: 10.1007/s00204-014-1319-2, 25107450 PMC4508371

[13] Xia L Park JH Biggs K Lee CG Liao L Shannahan JH. Compositional variations in metal nanoparticle components of welding fumes impact lung epithelial cell toxicity. J Toxicol Environ Health A. (2023) 86:735–57. doi: 10.1080/15287394.2023.2238209, 37485994

[14] Ellingsen DG Zibarev E Kusraeva Z Berlinger B Chashchin M Bast-Pettersen R . The bioavailability of manganese in welders in relation to its solubility in welding fumes. Environ Sci: Processes Impacts. (2013) 15:357–65. doi: 10.1039/C2EM30750B, 25208700

[15] Ghanem M Alleman LY Rousset D Perdrix E Coddeville P. Experimental factors influencing the bioaccessibility and the oxidative potential of transition metals from welding fumes. Environ Sci Process Impacts. (2024) 26:843–57. doi: 10.1039/D3EM00546A, 38597352

[16] Pajarillo E Nyarko-Danquah I Adinew G Rizor A Aschner M Lee E. Neurotoxicity mechanisms of manganese in the central nervous system. Adv Neurotoxicol. (2021) 5:215–38. doi: 10.1016/bs.ant.2020.11.003, 34263091 PMC8276947

[17] Antonini JM Santamaria AB Jenkins NT Albini E Lucchini R. Fate of manganese associated with the inhalation of welding fumes: potential neurological effects. Neurotoxicology. (2006) 27:304–10. doi: 10.1016/j.neuro.2005.09.001, 16219356

[18] Mauro M Crosera M Bovenzi M Adami G Baracchini E Maina G . In vitro meningeal permeation of MnFe2O4 nanoparticles. Chem Biol Interact. (2018) 293:48–54. doi: 10.1016/j.cbi.2018.07.020, 30053450

[19] Aschner M. Manganese: brain transport and emerging research needs. Environ Health Perspect. (2000) 108:429–32. doi: 10.1289/ehp.00108s3429, 10852840 PMC1637833

[20] Chen P Bornhorst J Aschner M. Manganese metabolism in humans. Front Biosci (Landmark Ed). (2018) 23:1655–79. doi: 10.2741/4665 29293455

[21] Roth J Ponzoni S Aschner M. Manganese homeostasis and transport. Met Ions Life Sci. (2013) 12:169–201. doi: 10.1007/978-94-007-5561-1_6, 23595673 PMC6542352

[22] Gunshin H Mackenzie B Berger UV Gunshin Y Romero MF Boron WF . Cloning and characterization of a mammalian proton-coupled metal-ion transporter. Nature. (1997) 388:482–8. doi: 10.1038/41343, 9242408

[23] Fitsanakis VA Zhang N Garcia S Aschner M. Manganese (Mn) and iron (Fe): interdependency of transport and regulation. Neurotox Res. (2010) 18:124–31. doi: 10.1007/s12640-009-9130-1, 19921534 PMC7909719

[24] Li GJ Zhang L-L Lu L Wu P Zheng W. Occupational exposure to welding fume among welders: alterations of manganese, iron, zinc, copper, and lead in body fluids and the oxidative stress status. J Occup Environ Med. (2004) 46:241–8. doi: 10.1097/01.jom.0000116900.49159.03, 15091287 PMC4126160

[25] Racette BA Searles Nielsen S Criswell SR Sheppard L Seixas N Warden MN . Dose-dependent progression of parkinsonism in manganese-exposed welders. Neurology. (2017) 88:344–51. doi: 10.1212/wnl.0000000000003533, 28031394 PMC5272970

[26] Kulshreshtha D Ganguly J Jog M. Manganese and movement disorders: a review. J Mov Disord. (2021) 14:93–102. doi: 10.14802/jmd.20123, 33819420 PMC8175808

[27] Huang C-C Weng Y-H Lu C-S Chu N-S Yen T-C. Dopamine transporter binding in chronic manganese intoxication. J Neurol. (2003) 250:1335–9. doi: 10.1007/s00415-003-0214-1, 14648150

[28] German Committee on Hazardous Substances. *Technical Rules for Hazardous Substances (TRGS) 900: Occupational Exposure Limits*. German Committee on Hazardous Substances (2025).

[29] American Conference of Governmental Industrial Hygienists. *TLV Documentation for Manganese, Elemental and Inorganic Compounds*. American Conference of Governmental Industrial Hygienists (2013).

[30] Halbach JH Cottam CM Tyler R. Occupational exposure to manganese from welding fumes during arc welding operations: data from the field. ACS Chem Health Saf. (2025) 32:366–77. doi: 10.1021/acs.chas.5c00048

[31] Knott P Csorba G Bennett D Kift R. Welding fume: a comparison study of industry used control methods. Safety. (2023) 9:42. doi: 10.3390/safety9030042

[32] Ellingsen DG Chashchin M Berlinger B Konz T Zibarev E Aaseth J . Biomarkers of iron status and trace elements in welders. J Trace Elem Med Biol. (2014) 28:271–7. doi: 10.1016/j.jtemb.2014.03.004, 24703374

[33] Lu L Zhang L-L Li GJ Guo W Liang W Zheng W. Alteration of serum concentrations of manganese, iron, ferritin, and transferrin receptor following exposure to welding fumes among career welders. Neurotoxicology. (2005) 26:257–65. doi: 10.1016/j.neuro.2004.09.001, 15713346 PMC4002285

[34] Baker MG Stover B Simpson CD Sheppard L Seixas NS. Using exposure windows to explore an elusive biomarker: blood manganese. Int Arch Occup Environ Health. (2016) 89:679–87. doi: 10.1007/s00420-015-1105-3, 26589320 PMC4829443

[35] Baker MG Simpson CD Stover B Sheppard L Checkoway H Racette BA . Blood manganese as an exposure biomarker: state of the evidence. J Occup Environ Hyg. (2014) 11:210–7. doi: 10.1080/15459624.2013.852280, 24579750 PMC3965573

[36] Casjens S Henry J Rihs H-P Lehnert M Raulf-Heimsoth M Welge P . Influence of welding fume on systemic iron status. Ann Occup Hyg. (2014) 58:1143–54. doi: 10.1093/annhyg/meu068, 25223225

[37] Schmied A Murawski A Kolossa-Gehring M Kujath P. Determination of trace elements in urine by inductively coupled plasma-tandem mass spectrometry - biomonitoring of adults in the German capital region. Chemosphere. (2021) 285:131425. doi: 10.1016/j.chemosphere.2021.131425, 34246933

[38] Blaszkewicz M Liesenhoff-Henze K. *Creatinine in urine [biomonitoring methods, 2010]*. The MAK-Collection for Occupational Health and Safety Part IV: Biomonitoring Methods, pp. 169–184. (2012).

[39] World Health Organization. Office of Occupational Health. Biological Monitoring of Chemical Exposure in the Workplace: Guidelines. Geneva: World Health Organization (1996). p. 300.

[40] German Research Foundation. *List of MAK and BAT Values 2025, Report 61: Maximum Concentrations at the Workplace and Assessment Values in Biological Material*. German Medical Science GMS Publishing House (2025).

[41] Heitland P Köster HD. Human biomonitoring of 73 elements in blood, serum, erythrocytes and urine. J Trace Elem Med Biol. (2021) 64:126706. doi: 10.1016/j.jtemb.2020.126706, 33352468

[42] Ellingsen DG Chashchin M Berlinger B Fedorov V Chashchin V Thomassen Y. Biological monitoring of welders’ exposure to chromium, molybdenum, tungsten and vanadium. J Trace Elem Med Biol. (2017) 41:99–106. doi: 10.1016/j.jtemb.2017.03.002, 28347469

[43] Rafiee A Wishart DS Yamamoto SS Pei L Quecke E Quémerais B. Assessing welding fume exposure in professional welders: an exploratory study of biomarkers and metabolomic profiles. Int J Hyg Environ Health. (2026) 271:114714. doi: 10.1016/j.ijheh.2025.114714, 41297109

[44] U.S. Occupational Safety and Health Administration. *Controlling Hazardous Fume and Gases during Welding*. OSHA Factsheet (2013).

[45] Cortes JB Sarazin P Dieme D Côté J Ouellet C El Majidi N . Biomonitoring of exposure to multiple metal components in urine, hair and nails of apprentice welders performing shielded metal arc welding (SMAW). Environ Res. (2023) 239:117361. doi: 10.1016/j.envres.2023.117361, 37844685

[46] Martinez-Morata I Sobel M Tellez-Plaza M Navas-Acien A Howe CG Sanchez TR. A state-of-the-science review on metal biomarkers. Curr Environ Health Rep. (2023) 10:215–49. doi: 10.1007/s40572-023-00402-x, 37337116 PMC10822714

[47] Ellingsen DG Dubeikovskaya L Dahl K Chashchin M Chashchin V Zibarev E . Air exposure assessment and biological monitoring of manganese and other major welding fume components in welders. J Environ Monit. (2006) 8:1078–86. doi: 10.1039/B605549D, 17240914

[48] Sadek AH Rauch R Schulz PE. Parkinsonism due to manganism in a welder. Int J Toxicol. (2003) 22:393–401. doi: 10.1177/109158180302200511, 14555414

[49] Rafiee A Ospina MB Pitt TM Quémerais B. Oxidative stress and DNA damage resulting from welding fumes exposure among professional welders: a systematic review and meta-analysis. Environ Res. (2022) 214:114152. doi: 10.1016/j.envres.2022.114152, 36041537

[50] Laohaudomchok W Lin X Herrick RF Fang SC Cavallari JM Christiani DC . Toenail, blood, and urine as biomarkers of manganese exposure. J Occup Environ Med. (2011) 53:506–10. doi: 10.1097/JOM.0b013e31821854da, 21494156 PMC3092003

[51] Ge X Wang F Zhong Y Lv Y Jiang C Zhou Y . Manganese in blood cells as an exposure biomarker in manganese-exposed workers healthy cohort. J Trace Elem Med Biol. (2018) 45:41–7. doi: 10.1016/j.jtemb.2017.09.016, 29173481

[52] Hoet P Vanmarcke E Geens T Deumer G Haufroid V Roels HA. Manganese in plasma: a promising biomarker of exposure to Mn in welders. A pilot study. Toxicol Lett. (2012) 213:69–74. doi: 10.1016/j.toxlet.2011.06.013, 21704687

[53] Karyakina NA Shilnikova N Farhat N Ramoju S Cline B Momoli F . Biomarkers for occupational manganese exposure. Crit Rev Toxicol. (2023) 52:636–63. doi: 10.1080/10408444.2022.2128718, 36705643

[54] Reiss B Simpson CD Baker MG Stover B Sheppard L Seixas NS. Hair manganese as an exposure biomarker among welders. Ann Occup Hyg. (2016) 60:139–49. doi: 10.1093/annhyg/mev064, 26409267 PMC4834832

[55] Wang D Du X Zheng W. Alteration of saliva and serum concentrations of manganese, copper, zinc, cadmium and lead among career welders. Toxicol Lett. (2008) 176:40–7. doi: 10.1016/j.toxlet.2007.10.003, 18054180 PMC3980858

[56] Bader M. *Manganese and its inorganic compounds – addendum for re-evaluation of the BAT value and evaluation of a BAR: Assessment Values in Biological Material – Translation of the German Version from 2011*. German Medical Science GMS Publishing House (2021).

[57] Persoons R Arnoux D Monssu T Culié O Roche G Duffaud B . Determinants of occupational exposure to metals by gas metal arc welding and risk management measures: a biomonitoring study. Toxicol Lett. (2014) 231:135–41. doi: 10.1016/j.toxlet.2014.09.008, 25223250

[58] Falcone LM Erdely A Kodali V Salmen R Battelli LA Dodd T . Inhalation of iron-abundant gas metal arc welding-mild steel fume promotes lung tumors in mice. Toxicology. (2018) 409:24–32. doi: 10.1016/j.tox.2018.07.007, 30055299 PMC6390845

[59] Michalek IM Martinsen JI Weiderpass E Hansen J Sparen P Tryggvadottir L . Heavy metals, welding fumes, and other occupational exposures, and the risk of kidney cancer: a population-based nested case-control study in three Nordic countries. Environ Res. (2019) 173:117–23. doi: 10.1016/j.envres.2019.03.023, 30903816

[60] Walters GO Miller FM Worwood M. Serum ferritin concentration and iron stores in normal subjects. J Clin Pathol. (1973) 26:770–2. doi: 10.1136/jcp.26.10.770, 4750458 PMC477879

[61] World Health Organization/Centers for Disease Control and Prevention. Assessing the Iron Status of Populations: Including Literature Reviews. Geneva: Switzerland (2007).

[62] Wang W Knovich MA Coffman LG Torti FM Torti SV. Serum ferritin: past, present and future. Biochim Biophys Acta. (2010) 1800:760–9. doi: 10.1016/j.bbagen.2010.03.011, 20304033 PMC2893236

[63] Badenhorst CE Forsyth AK Govus AD. A contemporary understanding of iron metabolism in active premenopausal females. Front Sports Act Living. (2022) 4:903937. doi: 10.3389/fspor.2022.903937, 35966107 PMC9366739

[64] Mintz J Mirza J Young E Bauckman K. Iron therapeutics in women’s health: past, present, and future. Pharmaceuticals (Basel). (2020) 13:13. doi: 10.3390/ph13120449, 33302392 PMC7762600

[65] Sorbie J Valberg LS Corbett WE Ludwig J. Serum ferritin, cobalt excretion and body iron status. Can Med Assoc J. (1975) 112:1173–8. 1125886 PMC1959113

[66] Tsuji M Hori H Koriyama C Tanaka R Isse T Ishihara Y . The effect of mask fit test on the association between the concentration of metals in biological samples and the results of time-weighted average personal exposure: a study on Japanese male welders. J Occup Health. (2023) 65:e12399. doi: 10.1002/1348-9585.12399, 37130744 PMC10154167

[67] Lehnert M Weiss T Pesch B Lotz A Zilch-Schöneweis S Heinze E . Reduction in welding fume and metal exposure of stainless steel welders: an example from the WELDOX study. Int Arch Occup Environ Health. (2014) 87:483–92. doi: 10.1007/s00420-013-0884-7, 23719851

