# Supplementary material for: Altered manganese and iron biomarkers in welders: a multi-element biomonitoring study across blood, serum and urine
Source: Front Public Health. 2026 Jun 15;14:1850447. doi: 10.3389/fpubh.2026.1850447 (PMC13310924; doi:10.3389/fpubh.2026.1850447)
Supplement: Supplementary file 1 [file Data_Sheet_1.DOCX]

Supplementary tables

**Table S1:** Tune modes, integration times, internal standards and applied limits of quantification (LOQ) for the
analysis of elements in urine, serum and blood samples.

| **Element** | **m/z** | **LOQ (μg/L)** | **Instrument mode** | **Integration time (s)** | **Internal standard** |
| --- | --- | --- | --- | --- | --- |
| *Matrix: Urine* | | | | | |
| Al | 27 | 2.0 | SQ, He | 3 | Sc |
| Ba | 137 | 0.05 | SQ, He | 1 | Tb |
| Be | 9 | 0.003 | SQ, No gas | 2 | Rh |
| Bi | 209 | 0.006 | SQ, No gas | 2 | Tb |
| Cd | 114🡪114 | 0.005 | MS/MS, O_2_ | 3 | Rh |
| Co | 59 | 0.003 | SQ, He | 1 | Rh |
| Cr | 52 | 0.08 | SQ, He | 1 | Rh |
| Cu | 63 | 0.55 | SQ, He | 1 | Rh |
| Fe | 56 | 1,56 | SQ, He | 1 | Rh |
| Li | 7 | 0.1 | SQ, No gas | 1 | Rh |
| Mn | 55 | 0.07 | SQ, He | 1 | Rh |
| Mo | 89🡪130 | 0.009 | MS/MS, O_2_ | 1 | Rh |
| Ni | 60 | 0.15 | SQ, He | 1 | Rh |
| Pb | 206+207+208 | 0.004 | SQ, No gas | 2 each isotope | Tb |
| Sb | 121 | 0.01 | SQ, He | 3 | Rh |
| Sr | 88 | 0.08 | SQ, He | 1 | Rh |
| V | 51🡪67 | 0.005 | MS/MS, O_2_ | 1 | Rh |
| *Matrix: Serum* | | | | | |
| Co | 59🡪59 | 0.009 | MS/MS, He | 1 | Rh |
| Cu | 63🡪63 | 0.3 | MS/MS, He | 1 | Rh |
| Fe | 56🡪56 | 18 | MS/MS, He | 1 | Rh |
| Mn | 55🡪55 | 0.09 | MS/MS, He | 1 | Rh |
| Zn | 66🡪66 | 1.0 | MS/MS, He | 1 | Rh |
| *Matrix: blood* | | | | | |
| Cd | 114🡪114 | 0.007 | O_2_, on mass | 3 | Rh |
| Co | 59🡪59 | 0.01 | MS/MS, He | 1 | Rh |
| Cu | 63🡪63 | 0.3 | MS/MS, He | 1 | Rh |
| Hg | 202🡪202 | 0.03 | MS/MS, He | 2 | Ir |
| Mn | 55🡪55 | 0.2 | MS/MS, He | 1 | Rh |
| Pb | 206🡪206+207🡪207+208🡪208 | 0.03 | MS/MS, He | 2 each isotope | Ir |
| Zn | 66🡪66 | 0.8 | MS/MS, He | 1 | Rh |

SQ – Single Quadrupole mode; MS/MS tandem mass spectrometry mode

**Table S2:** ICP-MS/MS operating conditions.

| RF Power | 1550 W |
| --- | --- |
| Nebulizer gas flow | 1 L/min |
| Auxiliary gas flow | 0.9 L/min |
| Plasma gas flow | 15 L/min |
| Nebulizer type | MicroMist |
| Spray chamber | Scott type |
| Replicates | 3 |
| Sweeps/ Replicate | 50 |
| Gas mode / cell gas flow (cell gas) | No gas / 0 mL/min (none) |
|  | He mode / 4.6 mL/min (Helium) |
|  | O_2_ mode / 0.5 mL/min (Oxygen) |

**Table S3:** Metals, ferritin, transferrin (Tf), transferrin saturation (TfS) and soluble transferrin receptor (sTfR) in serum and/or blood samples taken pre-shift or in the morning.

|  | | **Welders** | | | **Control group** | | |  |
| --- | --- | --- | --- | --- | --- | --- | --- | --- |
| **Parameter** | **N < LOQ W/C** | **GM** | **Median** | **IQR** | **GM** | **Median** | **IQR** | ***p*** |
| *In serum (µg/L)^a^* | | | | | | | | |
| Co | 0 / 0 | 0.127 | 0.133 | 0.114 – 0.146 | 0.161 | 0.173 | 0.124 – 0.198 | 0.06 |
| Cu | 0 / 0 | 930 | 900 | 865 – 988 | 813 | 787 | 719 – 912 | **0.02** |
| Fe | 0 / 0 | 970 | 1038 | 780 – 1163 | 957 | 959 | 698 – 1223 | 0.67 |
| Ferritin | 0 / 0 | 317 | 320 | 148 – 652 | 107 | 97 | 58.5 – 212.5 | **0.003** |
| Mn | 0 / 0 | 0.56 | 0.51 | 0.47 – 0.65 | 0.43 | 0.45 | 0.38 – 0.49 | **0.009** |
| sTfR (mg/L) | 0 / 0 | 2.28 | 2.22 | 2.09 – 2.54 | 2.57 | 2.56 | 2.30 – 2.87 | 0.06 |
| Tf (mg/dL) | 0 / 0 | 231 | 236 | 211 – 252 | 239 | 236 | 221 – 259 | 0.74 |
| TfS (%) | 0 / 0 | 29.8 | 34.2 | 20 – 39.3 | 28.3 | 27.7 | 19 – 36.7 | 0.53 |
| Zn | 0 / 0 | 833 | 829 | 810 – 924 | 773 | 765 | 692 – 843 | 0.085 |
| *In blood (µg/L)^a^* | | | | | | | | |
| Cd | 0 / 0 | 0.37 | 0.39 | 0.14 – 0.94 | 0.2 | 0.2 | 0.16 – 0.29 | 0.23 |
| Co | 0 / 0 | 0.101 | 0.102 | 0.09 – 0.11 | 0.127 | 0.119 | 0.099 – 0.175 | 0.085 |
| Cu | 0 / 0 | 793 | 795 | 776 – 828 | 782 | 778 | 713 – 840 | 0.56 |
| Hg | 1 / 1 | 0.39 | 0.4 | 0.26 – 0.87 | 0.31 | 0.35 | 0.13 – 1.07 | 0.78 |
| Mn | 0 / 0 | 9.70 | 10.0 | 7.90 – 13.3 | 7.82 | 7.85 | 6.77 – 10.1 | 0.06 |
| Pb | 0 / 0 | 16.5 | 16.6 | 10.3 – 22.4 | 13.3 | 10.9 | 8.36 – 15.6 | 0.21 |
| Zn | 0 / 0 | 6280 | 6210 | 6090 – 6560 | 6540 | 6720 | 6120 – 6970 | 0.21 |

N: number of samples, LOQ: limit of quantification, W: welders, C: control group, GM: geometric mean, IQR: interquartile range, *p*: *p*-value.

Significant p-values are given in bold.

^a^ Samples of 12 welders and 14 controls.

**Table S4:** Metals, metalloids, ferritin, transferrin (Tf), transferrin saturation (TfS) and soluble transferrin receptor (sTfR) in urine, serum and/or blood samples taken post-shift or in the afternoon.

|  | | **Welders** | | | **Control group** | | |  |
| --- | --- | --- | --- | --- | --- | --- | --- | --- |
| **Parameter** | **N < LOQ W/C** | **GM** | **Median** | **IQR** | **GM** | **Median** | **IQR** | ***p*** |
| *In urine (µg/g cr.)^a^* | | | | | | | | |
| Al | 0 / 12 | 3.68 | 2.98 | 2.30 – 5.92 | - | 1.3* | 0.75* – 1.9* | n.a. |
| Ba | 0 / 0 | 3.00 | 2.89 | 1.66 – 5.16 | 2.63 | 2.01 | 1.63 – 3.96 | 0.65 |
| Bi | 1 / 10 | 0.048 | 0.028 | 0.011 – 0.274 | - | 0.005* | 0.004* – 0.007 | n.a. |
| Cd | 0 / 0 | 0.15 | 0.15 | 0.08 – 0.27 | 0.12 | 0.11 | 0.08 – 0.18 | 0.69 |
| Co | 0 / 0 | 0.13 | 0.12 | 0.09 – 0.23 | 0.23 | 0.23 | 0.14 – 0.41 | 0.066 |
| Cr | 0 / 8 | 0.23 | 0.23 | 0.14 – 0.36 | - | 0.074* | 0.050* – 0.120 | n.a. |
| Cu | 0 / 0 | 7.41 | 7.63 | 6.28 – 9.43 | 8.40 | 8.63 | 6.86 – 10.41 | 0.20 |
| Fe | 0 / 0 | 11.4 | 13.9 | 4.90 – 23.2 | 3.32 | 3.16 | 2.55 – 3.83 | **<0.001** |
| Li | 0 / 0 | 20.7 | 19.4 | 15.1 – 27.6 | 21.4 | 19.8 | 18.1 – 27.0 | 0.61 |
| Mn | 3 / 14 | 0.13 | 0.09 | 0.05 – 0.41 | - | 0.05* | 0.03* – 0.06* | n.a. |
| Mo | 0 / 0 | 23.4 | 27.3 | 13.8 – 47.4 | 41.5 | 42.0 | 28.1 – 70.7 | **0.018** |
| Ni | 0 / 0 | 0.84 | 0.78 | 0.50 – 1.47 | 1.37 | 1.24 | 1.04 – 1.96 | 0.051 |
| Pb | 0 / 0 | 0.69 | 0.64 | 0.46 – 0.98 | 0.56 | 0.50 | 0.37 – 0.74 | 0.37 |
| Sb | 0 / 3 | 0.16 | 0.18 | 0.13 – 0.22 | 0.024 | 0.028 | 0.011* – 0.039 | **<0.001** |
| Sr | 0 / 0 | 114 | 125 | 74.0 – 152 | 124 | 127 | 106 – 166 | 0.65 |
| V | 0 / 4 | 0.060 | 0.056 | 0.041 – 0.097 | 0.018 | 0.027 | 0.005 – 0.041 | **<0.001** |
| *In urine (µg/L)^a^* | | | | | | | | |
| Al | 0 / 12 | 5.27 | 5.02 | 4.00 – 7.87 | - | <2.0 | <2.0 | n.a. |
| Ba | 0 / 0 | 4.30 | 5.74 | 2.15 – 8.1 | 2.27 | 2.17 | 1.13 – 4.24 | 0.10 |
| Bi | 1 / 10 | 0.068 | 0.028 | 0.011 – 0.387 | - | <0.006 | <0.006 – 0.008 | n.a. |
| Cd | 0 / 0 | 0.22 | 0.19 | 0.12 – 0.51 | 0.10 | 0.13 | 0.08 – 0.16 | **0.04** |
| Co | 0 / 0 | 0.19 | 0.26 | 0.11 – 0.33 | 0.21 | 0.24 | 0.13 – 0.3 | 0.85 |
| Cr | 0 / 8 | 0.34 | 0.35 | 0.19 – 0.67 | - | <0.08 | <0.08 – 0.15 | n.a. |
| Cu | 0 / 0 | 10.6 | 12.9 | 7.9 – 14.8 | 7.26 | 7.64 | 5.05 – 12.8 | 0.07 |
| Fe | 0 / 1 | 16.3 | 11.6 | 6.05 – 44.6 | 2.95 | 3.09 | 2.09 – 4.75 | **<0.001** |
| Li | 0 / 0 | 29.6 | 30.2 | 23.9 – 34.8 | 18.4 | 17.8 | 10.7 – 30.7 | 0.06 |
| Mn | 3 / 14 | 0.19 | 0.16 | <0.07 – 0.58 | <0.07 | <0.07 | <0.07 | n.a. |
| Mo | 0 / 0 | 33.5 | 35.2 | 18.5 – 54.2 | 35.8 | 40.0 | 17.9 – 63.2 | 0.81 |
| Ni | 0 / 0 | 1.21 | 1.31 | 0.73 – 1.84 | 1.18 | 1.18 | 0.83 – 1.88 | 0.94 |
| Pb | 0 / 0 | 0.99 | 1.14 | 0.53 – 1.63 | 0.48 | 0.36 | 0.27 – 1.0 | 0.06 |
| Sb | 0 / 3 | 0.23 | 0.260 | 0.15 – 0.36 | 0.021 | 0.024 | <0.01 – 0.044 | **<0.001** |
| Sr | 0 / 0 | 164 | 192 | 87.5 – 242 | 107 | 106 | 68.9 – 185 | 0.13 |
| V | 0 / 4 | 0.086 | 0.070 | 0.055 – 0.139 | 0.015 | 0.024 | <0.005 – 0.041 | **<0.001** |
| *In serum (µg/L)^b^* | | | | | | | | |
| Co | 0 / 0 | 0.130 | 0.133 | 0.119 – 0.144 | 0.176 | 0.188 | 0.137 – 0.229 | **0,011** |
| Cu | 0 / 0 | 950 | 917 | 882 – 995 | 846 | 834 | 719 – 960 | 0.052 |
| Fe | 0 / 0 | 885 | 860 | 792 – 1090 | 917 | 916 | 786 – 1060 | 0.81 |
| Ferritin | 0 / 0 | 303 | 303 | 149 – 587 | 110 | 98 | 62 – 225 | **0.008** |
| Mn | 0 / 0 | 0.61 | 0.55 | 0.49 – 0.77 | 0.44 | 0.45 | 0.40 – 0.48 | **<0.001** |
| sTfR (mg/L) | 0 / 0 | 2.27 | 2.27 | 2.03 – 2.50 | 2.58 | 2.52 | 2.36 – 2.98 | **0.046** |
| Tf (mg/dL) | 0 / 0 | 237 | 244 | 212 – 266 | 241 | 244 | 208 – 261 | 0.94 |
| TfS (%) | 0 / 0 | 26.5 | 26.2 | 21.7 – 33.5 | 27.0 | 26.3 | 19.0 – 32.1 | 1.0 |
| Zn | 0 / 0 | 714 | 702 | 673 – 750 | 720 | 744 | 654 – 791 | 0.54 |
| *In blood (µg/L)^b^* | | | | | | | | |
| Cd | 0 / 0 | 0.36 | 0.37 | 0.15 – 0.96 | 0.2 | 0.22 | 0.16 – 0.3 | 0.23 |
| Co | 0 / 0 | 0.103 | 0.102 | 0.094 – 0.112 | 0.135 | 0.136 | 0.100 – 0.169 | **0.016** |
| Cu | 0 / 0 | 807 | 806 | 764 – 851 | 797 | 784 | 726 – 870 | 0.77 |
| Hg | 1 / 1 | 0.40 | 0.38 | 0.25 – 0.99 | 0.39 | 0.46 | 0.23 – 1.11 | 0.85 |
| Mn | 0 / 0 | 9.6 | 10.1 | 8.03 – 13.5 | 7.86 | 8.22 | 6.64 – 10.2 | 0.098 |
| Pb | 0 / 0 | 16.1 | 15.9 | 10.3 – 21.0 | 13.5 | 11.2 | 7.96 – 16.6 | 0.41 |
| Zn | 0 / 0 | 6070 | 6170 | 5590 – 6530 | 6540 | 6820 | 6170 – 7060 | **0.035** |

N: number of samples, LOQ: limit of quantification, W: welders, C: control group, GM: geometric mean, IQR: interquartile range, *p*: *p*-value.

Significant p-values are given in bold. Be is not reported. In all samples, the Be concentration was below the LOQ.

^a^ Samples of 11 welders and 14 controls.

^b^ Samples of 12 welders and 13 controls.

^*^ Volume-based concentration of the spot urine sample is <LOQ.

**Table S5:** Metals, metalloids, ferritin, transferrin (Tf), transferrin saturation (TfS) and soluble transferrin receptor (sTfR) in urine, serum and/or blood samples taken post-shift depending on the use of powered air purifying respirators (PAPR) in comparison to the control group.

|  | **Welders using PAPR** | | | | **Welders without respiratory protection** | | | | **Control group** | | | |
| --- | --- | --- | --- | --- | --- | --- | --- | --- | --- | --- | --- | --- |
| **Parameter** | **N / N < LOQ** | **GM** | **Median** | **Range** | **N / N < LOQ** | **GM** | **Median** | **Range** | **N / N < LOQ** | **GM** | **Median** | **Range** |
| *In urine (µg/g cr.)* |  |  |  |  |  |  |  |  |  |  |  |  |
| Al | 6 / 0 | 4.2 | 2.86 | 2.03 – 17.9 | 5 / 0 | 3.14 | 2.98 | 2.23 – 5.92 | 14 / 12 | - | - | 0.56* – 8.17 |
| Ba | 6 / 0 | 2.51 | 2.79 | 1.24 – 5.16 | 5 / 0 | 3.72 | 2.89 | 1.66 – 7.96 | 14 / 0 | 2.63 | 2.01 | 1.28 – 11.3 |
| Bi | 6 / 1 | 0.024 | 0.022 | 0.002* – 0.27 | 5 / 0 | 0.109 | 0.162 | 0.006 – 1.29 | 14 / 10 | - | 0.005* | 0.002* – 0.031 |
| Cd | 6 / 0 | 0.15 | 0.12 | 0.04 – 0.74 | 5 / 0 | 0.16 | 0.15 | 0.10 – 0.27 | 14 / 0 | 0.12 | 0.11 | 0.05 – 0.26 |
| Co | 6 / 0 | 0.14 | 0.15 | 0.06 – 0.27 | 5 / 0 | 0.13 | 0.11 | 0.08 – 0.23 | 14 / 0 | 0.23 | 0.23 | 0.08 – 0.56 |
| Cr | 6 / 0 | 0.22 | 0.23 | 0.1 – 0.52 | 5 / 0 | 0.25 | 0.29 | 0.11 – 0.48 | 14 / 8 | - | 0.074* | 0.02* – 0.22 |
| Cu | 6 / 0 | 7.5 | 7.8 | 5.4 – 9.6 | 5 / 0 | 7.3 | 6.3 | 5.6 – 10.0 | 14 / 0 | 8.40 | 8.63 | 4.8 – 14.1 |
| Fe | 6 / 0 | 8.9 | 10.1 | 3.30 – 23.2 | 5 / 0 | 15.3 | 15.5 | 4.30 – 80.5 | 14 / 1 | 3.42 | 3.16 | 1.82* – 15.7 |
| Li | 6 / 0 | 26.9 | 25.3 | 18.1 – 54.4 | 5 / 0 | 15.1 | 15.1 | 12.4 – 19.4 | 14 / 0 | 21.4 | 19.8 | 10.8 – 36.5 |
| Mn | 6 / 3 | 0.06 | 0.08 | 0.02* – 0.12 | 5 / 0 | 0.33 | 0.41 | 0.05 – 2.23 | 14 / 14 | - | 0.05* | 0.02* – 0.08* |
| Mo | 6 / 0 | 34 | 38.4 | 14.9 – 51.3 | 5 / 0 | 14.9 | 13.8 | 7.5 – 27.3 | 14 / 0 | 41.5 | 42.0 | 15.0 – 80.8 |
| Ni | 6 / 0 | 0.99 | 0.83 | 0.48 – 3.15 | 5 / 0 | 0.69 | 0.69 | 0.35 – 1.47 | 14 / 0 | 1.37 | 1.24 | 0.51 – 4.48 |
| Pb | 6 / 0 | 0.66 | 0.6 | 0.28 – 2.28 | 5 / 0 | 0.73 | 0.64 | 0.52 – 1.49 | 14 / 0 | 0.56 | 0.50 | 0.17 – 3.25 |
| Sb | 6 / 0 | 0.14 | 0.17 | 0.05 – 0.18 | 5 / 0 | 0.19 | 0.22 | 0.12 – 0.27 | 14 / 3 | 0.024 | 0.028 | 0.004* – 0.12 |
| Sr | 6 / 0 | 126 | 136 | 62 – 204 | 5 / 0 | 102 | 125 | 61 – 144 | 14 / 0 | 124 | 127 | 52 – 208 |
| V | 6 / 0 | 0.051 | 0.045 | 0.039 – 0.105 | 5 / 0 | 0.072 | 0.087 | 0.039 – 0.101 | 14 / 4 | 0.018 | 0.027 | 0.002* – 0.068 |
| Al | 6 / 0 | 5.03 | 4.81 | 2.35 – 10.7 | 5 / 0 | 5.56 | 5.31 | 3.06 – 8.29 | 14 / 12 | - | <2.0 | <2 – 7.2 |
| Ba | 6 / 0 | 3.01 | 3.95 | 0.57 – 8.1 | 5 / 0 | 6.6 | 6.26 | 2.27 – 15.4 | 14 / 0 | 2.27 | 2.17 | 0.86 – 10.6 |
| Bi | 6 / 1 | 0.029 | 0.026 | 0.003 – 0.39 | 5 / 0 | 0.19 | 0.35 | 0.01 – 2.61 | 14 / 10 | - | <0.006 | <0.006 – 0.016 |
| Cd | 6 / 0 | 0.18 | 0.15 | 0.02 – 1.14 | 5 / 0 | 0.28 | 0.33 | 0.14 – 0.51 | 14 / 0 | 0.10 | 0.13 | 0.03 – 0.28 |
| Co | 6 / 0 | 0.17 | 0.21 | 0.05 – 0.39 | 5 / 0 | 0.22 | 0.26 | 0.11 – 0.34 | 14 / 0 | 0.21 | 0.24 | 0.061 – 0.51 |
| Cr | 6 / 0 | 0.26 | 0.28 | 0.09 – 0.81 | 5 / 0 | 0.45 | 0.64 | 0.19 – 0.85 | 14 / 8 | - | <0.08 | <0.08 – 0.28 |
| Cu | 6 / 0 | 8.98 | 11.4 | 2.47 – 15.2 | 5 / 0 | 13.0 | 13.7 | 7.9 – 17.8 | 14 / 0 | 7.26 | 7.64 | 2.08 – 14.2 |
| Fe | 6 / 0 | 10.6 | 8.2 | 3.9 – 44.6 | 5 / 0 | 27.2 | 21.3 | 6.1 – 189 | 14 / 1 | 2.95 | 3.09 | <1.6 – 8.0 |
| Li | 6 / 0 | 32.2 | 33.4 | 11.6 – 85.4 | 5 / 0 | 26.8 | 27 | 20.7 – 34.6 | 14 / 0 | 18.4 | 17.8 | 8.0 – 41.7 |
| Mn | 6 / 3 | 0.07 | 0.07 | 0.04 – 0.18 | 5 / 0 | 0.59 | 0.58 | 0.08 – 5.25 | 14 / 14 | <0.07 | <0.07 | <0.07 |
| Mo | 6 / 0 | 40.7 | 50.2 | 17.3 – 79 | 5 / 0 | 26.5 | 29.1 | 17.6 – 38.2 | 14 / 0 | 35.8 | 40 | 13.2 – 110 |
| Ni | 6 / 0 | 1.19 | 1.05 | 0.67 – 3.65 | 5 / 0 | 1.23 | 1.51 | 0.48 – 2.06 | 14 / 0 | 1.18 | 1.18 | 0.31 – 3.58 |
| Pb | 6 / 0 | 0.79 | 0.84 | 0.18 – 3.52 | 5 / 0 | 1.3 | 1.36 | 0.87 – 2.08 | 14 / 0 | 0.48 | 0.36 | 0.11 – 2.86 |
| Sb | 6 / 0 | 0.16 | 0.210 | 0.07 – 0.27 | 5 / 0 | 0.35 | 0.36 | 0.21 – 0.58 | 14 / 3 | 0.021 | 0.024 | <0.01 – 0.07 |
| Sr | 6 / 0 | 151 | 175 | 59 – 320 | 5 / 0 | 181 | 192 | 83 – 338 | 14 / 0 | 107 | 106 | 22.3 – 305 |
| V | 6 / 0 | 0.062 | 0.061 | 0.04 – 0.11 | 5 / 0 | 0.129 | 0.139 | 0.07 – 0.23 | 14 / 4 | 0.015 | 0.024 | <0.005 – 0.06 |
| Co | 7 / 0 | 0.136 | 0.14 | 0.117 – 0.149 | 5 / 0 | 0.12 | 0.126 | 0.094 – 0.153 | 13 / 0 | 0.176 | 0.188 | 0.091 – 0.26 |
| Cu | 7 / 0 | 1000 | 980 | 888 – 1255 | 5 / 0 | 884 | 880 | 850 – 920 | 13 / 0 | 846 | 834 | 680 – 1170 |
| Fe | 7 / 0 | 838 | 830 | 490 – 1270 | 5 / 0 | 954 | 960 | 800 – 1140 | 13 / 0 | 917 | 960 | 477 – 2000 |
| Ferritin | 7 / 0 | 215 | 193 | 89 – 1111 | 5 / 0 | 491 | 570 | 209 – 752 | 13 / 0 | 110 | 98 | 27 – 303 |
| Mn | 7 / 0 | 0.53 | 0.53 | 0.41 – 0.66 | 5 / 0 | 0.74 | 0.8 | 0.5 – 1.16 | 13 / 0 | 0.44 | 0.45 | 0.32 – 0.54 |
| sTfR (mg/L) | 7 / 0 | 2.3 | 2.32 | 2.01 – 2.94 | 5 / 0 | 2.23 | 2.26 | 1.74 – 2.85 | 13 / 0 | 2.58 | 2.52 | 2.02 – 3.40 |
| Tf (mg/dL) | 7 / 0 | 234 | 249 | 195 – 270 | 5 / 0 | 241 | 238 | 211 – 272 | 13 / 0 | 241 | 244 | 198 – 314 |
| TfS (%) | 7 / 0 | 25.4 | 24.8 | 16.1 – 39.3 | 5 / 0 | 28.1 | 26.9 | 21.6 – 36.2 | 13 / 0 | 27.0 | 26.3 | 13.2 – 71.3 |
| Zn | 7 / 0 | 709 | 699 | 670 – 770 | 5 / 0 | 722 | 720 | 640 – 850 | 13 / 0 | 720 | 744 | 589 - 812 |
| Cd | 7 / 0 | 0.35 | 0.58 | 0.07 – 1.74 | 5 / 0 | 0.44 | 0.55 | 0.14 – 1.19 | 13 / 0 | 0.2 | 0.22 | 0.09 – 0.38 |
| Co | 7 / 0 | 0.104 | 0.101 | 0.087 – 0.132 | 5 / 0 | 0.102 | 0.103 | 0.082 – 0.135 | 13 / 0 | 0.135 | 0.136 | 0.092 – 0.23 |
| Cu | 7 / 0 | 815 | 814 | 756 – 909 | 5 / 0 | 797 | 802 | 734 – 880 | 13 / 0 | 797 | 784 | 681 – 952 |
| Hg | 7 / 1 | 0.31 | 0.37 | <0.03 – 3.17 | 5 / 0 | 0.57 | 0.46 | 0.23 – 1.6 | 13 / 1 | 0.39 | 0.46 | <0.03 – 1.9 |
| Mn | 7 / 0 | 8.13 | 8.19 | 4.32 – 14.0 | 5 / 0 | 12.1 | 11.82 | 9.94 – 14.4 | 13 / 0 | 7.86 | 8.22 | 4.63 – 10.7 |
| Pb | 7 / 0 | 13.4 | 11 | 5.30 – 52.7 | 5 / 0 | 20.6 | 20 | 14.4 – 36.8 | 13 / 0 | 13.5 | 11.2 | 5.3 – 68.7 |
| Zn | 7 / 0 | 6000 | 6140 | 5280 – 6590 | 5 / 0 | 6170 | 6190 | 5540 – 6810 | 13 / 0 | 6540 | 6820 | 5020 – 7470 |

N: number of samples, LOQ: limit of quantification, GM: geometric mean, IQR: interquartile range,

* Volume-based concentration of the spot urine sample is <LOQ.

**Table S6:** Metals and metalloids in urine, serum and blood samples of the control group compared with data of inhabitants in northern Germany and Biological Reference Values (BAR).

|  | Control group | | BAR^a^ | Inhabitants of northern Germany^b^ | | |
| --- | --- | --- | --- | --- | --- | --- |
| Parameter | Mean | Range |  | Mean | Range | 95^th^ percentile |
| *In urine (µg/L)* | | | | | | |
| Al | - | <2 – 7.2 | - | 3 | <2 – 10.9 | 6.6 |
| Ba | 3.03 | 0.86 – 10.6 | 10 | 2.9 | 0.1 – 71 | 11 |
| Bi | - | <0.006 – 0.016 | - | 0.01 | <0.01 – 0.13 | 0.028 |
| Cd | 0.12 | 0.03 – 0.28 | 0.8^c^ | 0.23 | <0.1 – 2.4 | 0.58 |
| Co | 0.229 | 0.061 – 0.51 | 1.5 | 0.34 | 0.026 – 2.6 | 1.3 |
| Cr | - | <0.08 – 0.28 | 0.6 | 0.17 | <0.1 – 1.1 | 0.35 |
| Cu | 8.29 | 2.08 – 14.2 | - | 6.7 | 1 – 19 | 15 |
| Fe | 3.39 | <1.56 – 8.0 | - | 8.2 | <1 – 103 | 18 |
| Li | 21.1 | 8.0 – 41.7 | 100 | 27.6 | 2 – 416 | 103 |
| Mn | - | <0.07 | - | 0.06 | <0.06 – 0.33 | 0.12 |
| Mo | 45.3 | 13.2 – 110 | 150 | 30 | 2 – 248 | 78 |
| Ni | 1.43 | 0.31 – 3.58 | 3 | 1.35 | <0.4 – 6.2 | 4 |
| Pb | 0.71 | 0.11 – 2.86 | - | 0.46 | 0.06 – 2.7 | 1 |
| Sb | 0.029 | <0.01 – 0.07 | 0.2 | 0.03 | <0.015 – 0.15 | 0.08 |
| Sr | 132.5 | 22.3 – 305 | - | 112 | 14 – 777 | 286 |
| V | 0.026 | <0.005 – 0.06 | 0.15 | 0.055 | <0.035 – 0.29 | 0.13 |
| In serum (µg/L) | | | | | | |
| Co | 0.18 | 0.091 – 0.26 | - | 0.15 | 0.04 – 0.77 | 0.387 |
| Cu | 856 | 680 – 1170 | - | 1188 | 560 – 2280 | 1949 |
| Fe | 983 | 477 – 2000 | - |  |  |  |
| Mn | 0.44 | 0.32 – 0.54 | - | 0.48 | 0.29 – 0.63 | 0.6 |
| Zn | 724 | 589 – 812 | - | 911 | 605 – 1348 | 1110 |
| In blood (µg/L) | | | | | | |
| Cd | 0.22 | 0.09 – 0.38 | 1^c^ | 0.55 | <0.1 – 7.8 | 1.7 |
| Co | 0.14 | 0.092 – 0.23 | - | 0.16 | 0.04 – 0.72 | 0.39 |
| Cu | 802 | 681 – 952 | - | 951 | 594 – 1689 | 1360 |
| Hg | 0.7 | <0.03 – 1.9 | - | 0.65 | 0.03 – 3.5 | 1.7 |
| Mn | 8.09 | 4.63 – 10.7 | 15 | 8.9 | 3.6 – 20.2 | 13.5 |
| Pb | 19.5 | 5.3 – 68.7 | 40^d^ | 13 | 2.9 – 55.8 | 26.3 |
| Zn | 6580 | 5020 – 7470 | - | 5660 | 3850 – 7520 | 6730 |

| ^a^ German Research Foundation, 2025. List of MAK and BAT Values 2025, German Medical Science. https://doi.org/10.34865/mbwl_2025_eng.  ^b^ P. Heitland, H. D. Köster, 2021. Human biomonitoring of 73 elements in blood, serum, erythrocytes and urine. J. Trace Elem. Med. Biol. 64, 126706.  https://doi.org/10.1016/j.jtemb.2020.126706.  ^c^ evaluated for non-smokers |
| --- |

^d^ for men

Supplementary figures

**Figure S1:** Scatterplots of correlations between manganese, vanadium and ferritin in welders. Each panel shows a Spearman’s rank correlation between two variables as indicated. Correlation coefficients (ρ) and p-values are displayed in the respective panels A-F. Each point represents one participant.
